# Schiff Bases: A Short Survey on an Evergreen Chemistry Tool

**DOI:** 10.3390/molecules181012264

**Published:** 2013-10-08

**Authors:** Wenling Qin, Sha Long, Mauro Panunzio, Stefano Biondi

**Affiliations:** 1 Dipartimento di Chimica “G. Ciamician” Via Selmi 2, Bologna 40126, Italy; E-Mails: wenling.qin2@unibo.it (W.L.Q.); sha.long2@unibo.it (S.L.); 2 ISOF-CNR Dipartimento di Chimica “G. Ciamician” Via Selmi 2, Bologna 40126, Italy; 3 Allecra Therapeutics SAS, 13, Rue du Village Neuf, Saint Louis 68300, France

**Keywords:** imines, Schiff bases, metallo-imines, salen complexes, bio-active-imines

## Abstract

The review reports a short biography of the Italian naturalized chemist Hugo Schiff and an outline on the synthesis and use of his most popular discovery: the imines, very well known and popular as Schiff Bases. Recent developments on their “metallo-imines” variants have been described. The applications of Schiff bases in organic synthesis as partner in Staudinger and hetero Diels-Alder reactions, as “*privileged*” ligands in the organometallic complexes and as biological active Schiff intermediates/targets have been reported as well.

## 1. Introduction

### 1.1. Ugo Schiff (Frankfurt, 26 April 1834-Florence, 8 September 1915): A Brief Biography

**Figure 1:**
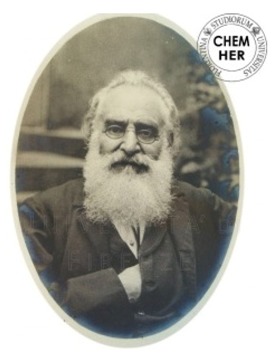
A portrait of Hugo Schiff.

Ugo (Hugo) Joseph Schiff (Figure 1), one of the founders of modern chemistry, was born in Frankfurt on the 26 April 1834, into a wealthy Jewish family of merchants, Joseph Moses Schiff (1784–1852) and Henriette Trier (1798–1888). 

**Figure 2:**
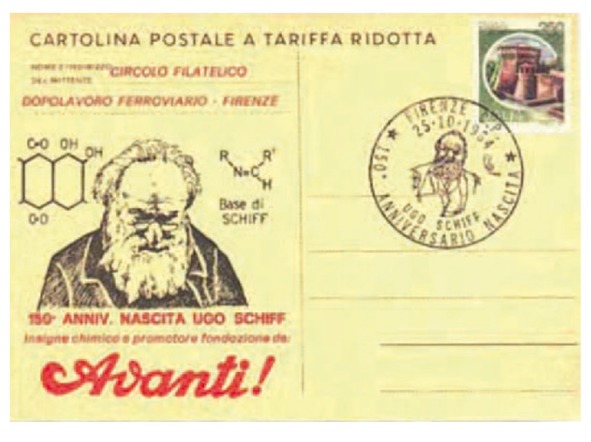
A commemorative Postal card celebrating the 150th anniversary of Ugo Schiff’s birth.

He was the eighth son out of ten, of which only four, Moritz, Hugo, Bertha and Clementine reached adulthood [1]. He studied chemistry and physics in Frankfurt with Professors Böetteger and Löwe, and continued his studies in Göttingen, where he got his degree in 1857 under the supervision of professor Wölher. Professor Wölher was, in turn, student of Berzelius in Stockolm and was the first chemist to synthesize urea, an organic molecule, starting from inorganic compounds: the birth of modern organic chemistry is taught to start from this experiment, which, once and for all, excluded the presence of “*vis-vitalis*” (vital strength residing in the organic matter) demonstrating that there is no metaphysical difference between organic and inorganic substances. This was the origin of organic chemistry and the beginning of a new type of scientific research. Professor Schiff was used to say to his pupils: “*Remember that you descend from Berzelius, because Berzelius taught Chemistry to the old Wöhler and the old Wöhler taught me*.” [2]. In 1856 Ugo Schiff moved out of Germany because of his Jewish origins and political ideas and spent six years in Bern before reaching Italy where he remained for the rest of his career. On this base Professor Schiff must be fully considered an Italian Chemist. Schiff retained his liberal views and was a cofounder of the socialist Italian newspaper *L’Avanti* in 1894 (Figure 2).

**Figure 3:**
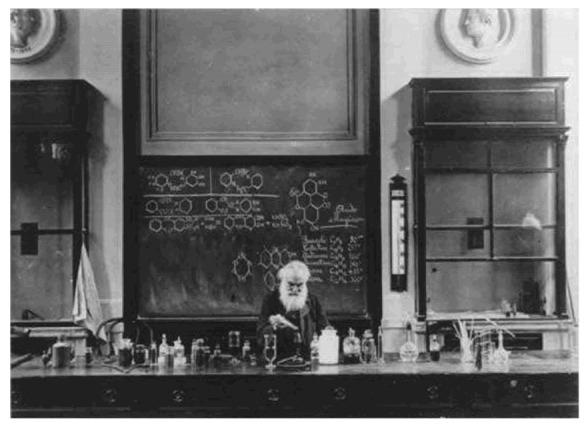
Hugo Schiff, 24 April 1915.

He started by teaching chemistry as assistant professor at the University of Pisa and in 1864 was nominated professor at the *Regio Istituto di Studi Superiori Pratici e di Perfezionamento* of Florence, the future University of Florence, where he was the first chemistry teacher. Between 1864 and 1915, Ugo Schiff spent his entire career in Florence and continued teaching until 1915, the year of his death (Figure 3).

He devoted his interest to organic and inorganic chemistry, physical and analytical chemistry, mineralogy, and natural substances. His studies on Schiff bases, target of this Review, are very popular. The name “Organic Bases” appears in a paper entitled “*A New Series of Organic Bases*” (“*Eine neue Reihe organischer Basen*”) [3]. The designation of these compounds as bases, although they are not used as bases in the conventional sense, has persisted up to the present time [4]. In the meantime, boric ethers, glucosides, arbutin, tannin and gallic acid, aromatic carboxylic acids and asparagine, urea and its derivatives were also studied by Schiff. He developed the analytical methodology, later used by Sörensen, to determine amino acids in urine, and he devised the Schiff fuchsin aldehyde test [5], still in use nowadays [6]. Thionyl chloride must also be cited as one of his important discoveries [7].

### 1.2. Schiff Bases: Physical-Chemical Properties

Imines, known even as azomethines or Schiff bases [3,8,9,10,11,12,13,14] are compounds that are represented by the general formula R_3_R_2_C=NR_1_. The substituents R_2_ and R_3_ may be alkyl, aryl, heteroaryl, hydrogen. The substituent at the *N-*imino (C=N) may be alkyl, aryl, heteroaryl, hydrogen or metallo (usually Si, Al, B, Sn). The physical properties and reactivity of imines are and continue to be studied by more than a hundred years [15]. Physical-chemical properties (IR, Raman, ^1^H-NMR, ^13^C-NMR) of a large variety of Schiff bases are easily found in any current dedicated textbook.

## 2. Preparations of Imines

### 2.1. Preparation of N-Aryl or Alkyl Substituted Imines

#### 2.1.1. Reaction of Aldehydes and Ketones with Amines

**Scheme 1:**
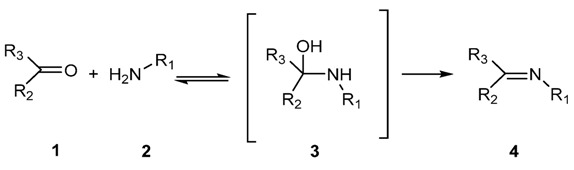
Schiff reaction for the preparation of imines.

The most common method for preparing imines is the original reaction discovered by Schiff [3,5,11,16,17]. Basically it consists in the reaction of an aldehyde (respectively a ketone) with a primary amine and elimination of one water molecule (Scheme 1). This reaction can be accelerated by acid catalysis and is generally carried out by refluxing a mixture of a carbonyl compound **1** and an amine **2**, in a Dean Stark apparatus in order to remove the water. This removal is important as the conversion of aminal **3** into the imine **4** is reversible (Scheme 1). From this point several dehydrating agents have been successfully used including sodium sulphate and molecular sieves [18]. Alternatively, some *in situ* methods, involving dehydrating solvents such as tetramethyl orthosilicate or trimethyl orthoformate, have been reported as well [19,20]. As far as the use of acid catalyst is required [21,22,23,24,25,26,27], mineral acids, like H_2_SO_4_ or HCl, organic acids such as *p*-toluene sulphonic acids or pyridinium *p*-toluenesulphonate, acid resin, montmorillonite or even Lewis acids like ZnCl_2_, TiCl_4_, SnCl_4_, BF_3_Et_2_O, MgSO_4_, Mg(ClO_4_)_2_, *etc*., have been reported.

**Scheme 2:**
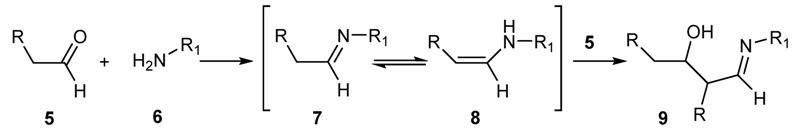
Aldol like condensation of aliphatic aldehydes.

In the course of the preparation of imines, if aliphatic aldehydes are used, a known competitive reaction, due to the formation of a condensation product arising from an aldol type reaction, can occur as well (Scheme 2).

Aliphatic ketones react with amines to form imines more slowly than aldehydes, therefore, higher reaction temperatures and longer reaction time are required. Acid catalysts and water removal from the reaction mixture can significantly increase the reaction yields, which can reach 80%–95% values. Aromatic ketones are less reactive than aliphatic ones and require harsh conditions to be converted into imines [28]. Recently, several new techniques to produce imines have been published, including solvent-free, clay, microwave irradiation, water suspension medium, liquid crystals, molecular sieves, infrared and ultrasound irradiation [29,30,31,32,33,34,35,36].

#### 2.1.2. Aerobic Oxidative Synthesis in the Preparation of Schiff’s Bases

**Scheme 3:**
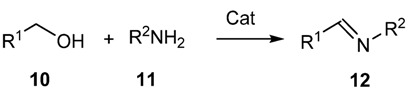
Oxidative synthesis of imines from alcohols and amines.

**Scheme 4:**
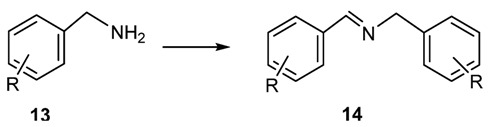
Oxidative synthesis of imines from amines.

Since aldehydes and ketones are mostly obtained from the corresponding alcohols via oxidative process, a straightforward preparation of imines from amines and alcohols, through tandem oxidative processes, have recently been developed (Scheme 3 and Scheme 4) [37,38,39,40,41,42,43,44].

Following this general approach a mild and efficient method of amine oxidation has been reported by Huang and Largeron (Scheme 4) [39,45].

#### 2.1.3. Addition of Organometallic Reagents to Cyanides

**Scheme 5:**
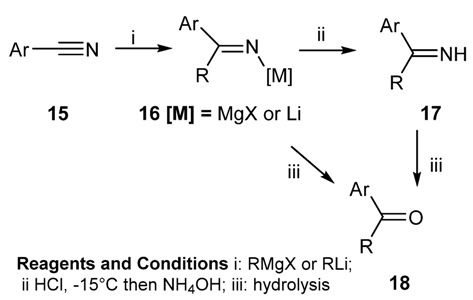
Addition of organometallic reagents to cyanides.

Addition of Grignard or organolithium reagents to aryl cyanides can lead to unsubstituted ketimines which, in turn, can be elaborated to the corresponding ketones depending on the hydrolysis conditions used to decompose the metallo imine intermediate **16** (Scheme 5). The reaction has also been extended to aliphatic cyanides [46], producing very high yields of ketimines, provided that the Mg-imine intermediate is treated with anhydrous methanol [47]. The use of heteroaryl lithium reagents affording the corresponding ketimines has also been reported [48].

#### 2.1.4. Reaction of Phenols and Phenol-Ethers with Nitriles

**Scheme 6:**
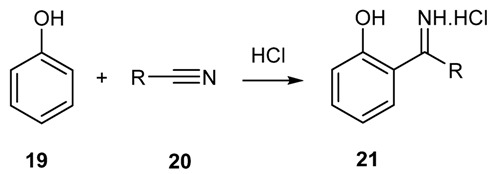
Synthesis of ketimines from phenols and nitriles.

Alkyl and aryl cyanides react smoothly with phenols and their ethers producing ketimines in very good yields in the presence of an acid catalyst (Scheme 6) [49,50,51]. The reaction is performed by mixing the nitrile and phenol in ether and saturating the solution with gaseous HCl, whereas, for less reactive phenols, ZnCl_2_ must be used.

#### 2.1.5. Reaction of Metal Amides

Ketimine has been produced by the addition reactions of alkali metal (or calcium amine salts) to aromatic ketones [Equation (1)]. The scope of this reaction has been widely extended [52]:

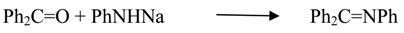
(1)


**Scheme 7:**
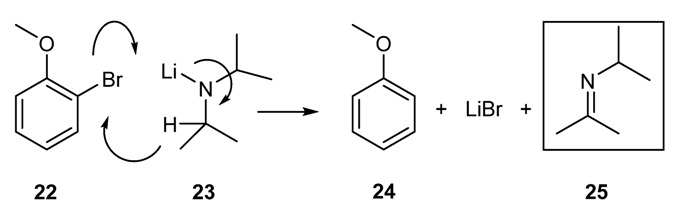
Oxidation of metal amines to imines by 2-bromoanisole.

An interesting reaction is the oxidation of metalloamines bearing an α-hydrogen by 2-bromoanisole [53] to yield imines (Scheme 7).

#### 2.1.6. Other Methodologies

**Scheme 8:**
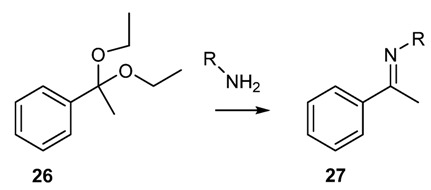
Synthesis of ketimines from ketals.

Ketimine can be prepared in high yield using aryl ketone diethyl ketals and arylamines, while alkylamines give only low yields (Scheme 8) [54]. Similarly, imines can react with higher boiling point amines to give the exchange products. The latter can be distilled driving the equilibrium towards the formation of the desired product [55].

**Scheme 9:**
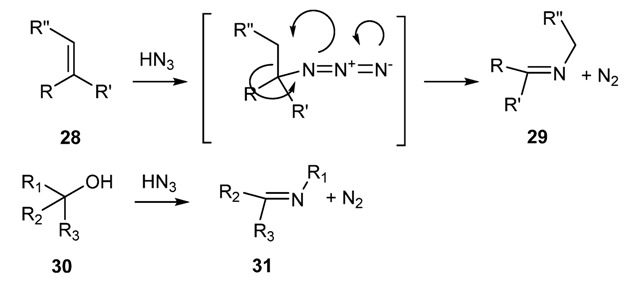
Reaction of olefins and tertiary alcohols with hydrazoic acid.

Olefins and tertiary alcohols can be converted into ketimines [56] by reaction of hydrazoic acid in sulfuric acid (Scheme 9).

**Scheme 10:**
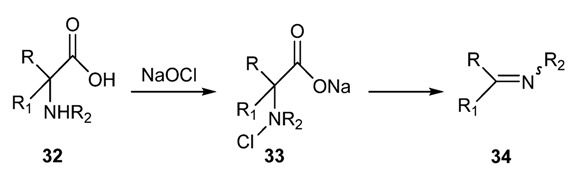
Conversion of α-amino acids into imines.

Imines can also be formed by reaction of amino acids with sodium hypochlorite (Scheme 10). The first step of this reaction is the formation of a chloramine intermediate that gives rise to the imine via elimination of carbon dioxide and sodium chloride [57].

### 2.2. Preparation of *N*-Metallo-Imines as Stable Synthetic Equivalents of *N*-Unsubstituted Schiff Bases [58]

*N*-metallo-imines constitute a young family of organometallic compounds congeners of Schiff bases [59]. They have been found synthetic applications in the last few decades as relatively stable analogues of the corresponding Schiff bases. Their elaborations to azadiene have been fully explored by the Barluenga [60,61,62], Ghosez [63,64] and Panunzio groups [65,66,67,68,69]. Generally speaking, they are monomeric compounds reasonably stable under anhydrous conditions. Since the metal-nitrogen bond is easily hydrolysed, the *N*-metalloimines may be considered a protected, stabilized form of the corresponding elusive imines of ammonia, which are known to be very unstable readily trimerizing to triazines [11]. Although some metalloimines, e.g., the silylimines of certain aldehydes, can be isolated in a pure form by distillation under reduced pressure, for synthetic purposes it is in general more convenient to prepare them *in situ* just before the use. In this case it is possible to ascertain their structure by a combined use of IR, ^1^H-NMR, ^13^C-NMR and mass spectroscopic techniques.

#### 2.2.1. Preparation of Certain *N*-Metallo Imines (Metallo = B, Al, Si, Sn) [70]

##### 2.2.1.1. Preparation of *N*-Boryl [70,71,72,73] and N-Aluminium Imines [74,75,76,77,78]: Addition of an Organometallic Reagents or a Metallo Hydride to a Nitrile [59,79,80,81,82]

**Scheme 11:**
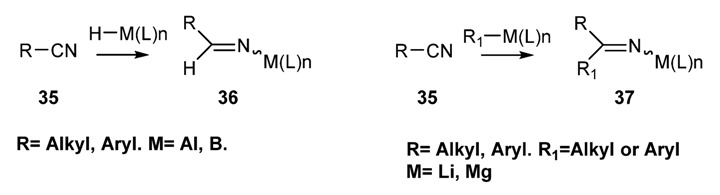
Preparation of *N*-metalloimines from nitriles.

Scheme 11 illustrates the general procedure used to prepare metalloimines starting from nitriles **35** and a suitable organometallic reagent either by hydrometallation to give compounds of general formula **36** or by alkylation to give compounds of general formula **37**.

##### 2.2.1.2. Preparation of *N*-Silylimines

###### 2.2.1.2.1. Via Reaction of the Hexalkyldisilylamide of Group I Metals (Li, Na, K) with an Aldehyde or a Nonenolizable Ketones [83,84,85,86]

**Scheme 12:**
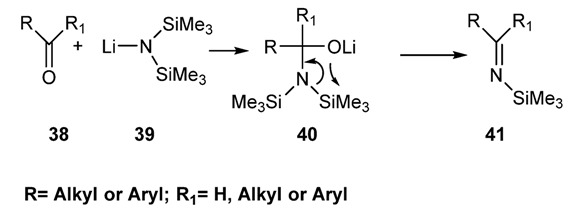
Preparation of *N-*silylimines via reaction of lithium hexalkyldisilylamide.

Among different metalloimines, *N-*trialkylsilyl imines must be considered the most popular and the most used intermediates in the preparation of nitrogen containing organic compounds, with special emphasis to the potentially bioactive ones [67,86]. Silyl imines have been prepared, for the first time, by Rochow [84] starting from aromatic aldehydes and nonenolizable ketones by treatment of the carbonyl compounds with one equivalent of lithium hexamethyldisilylamide in tetrahydrofuran [86]. The reaction proceeds by an addition-elimination sequence probably involving a four centers cyclic transition state (Scheme 12).

**Scheme 13:**
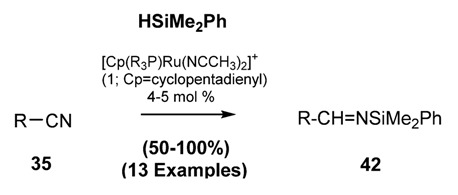
*N*-alkylsilyl imines via hydrosilylation of nitrile.

Ketones, bearing a hydrogen atom in α-position to the carbonyl group, failed to produce the silylimines since in this case the strongly basic organometallic reagent attacks an α-hydrogen affording the corresponding lithium enolate. Enolizable aldehydes were supposed to behave in the same way [85]. This notwithstanding the preparation of such silyl imines is easier than one might expect [87]. Few competitive methods, to the above cited, have been reported in the last few years on the preparation of *N*-alkylsilyl imines. Very recently Nikonov and co-workers [88] reported an elegant preparation of *N*-silyl-aldimines **42** via a chemoselective hydrosilylation of nitriles **35** catalysed by ruthenium complex (Scheme 13).

###### 2.2.1.2.2. Preparation of *N*-Silylimines via Base-Induced Elimination of Vicinal Substituent from *N*-Silyl Amines [89,90]

**Scheme 14:**
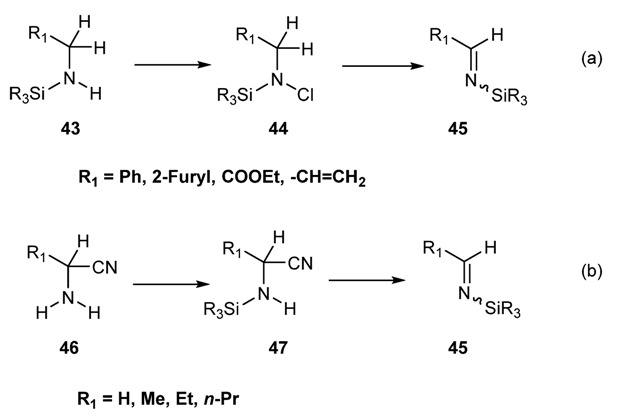
Formation of silylimines via elimination of vicinal groups. (**a**) from *N*-chloro silylamines; (**b**) from α-cyano silylamines.

In analogy of classical preparation of Schiff Bases silyl-imines **45** may be prepared by elimination of vicinal substituents as shown in (Scheme 14).

##### 2.2.1.3. Preparation of *N*-tin-Imines via Reaction of Carbonyl Compounds with Tris(trimethylstannyl)amine [91]

**Scheme 15:**
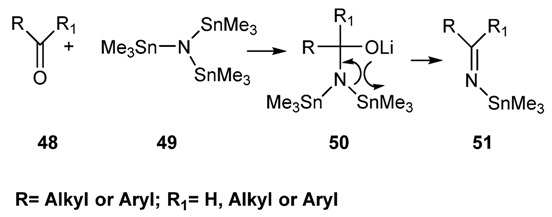
Synthesis of *N*-tin imines.

This method allows the preparation of tin imines from enolizable and non enolizable aldehydes and ketones, in good yield and under very mild conditions (Scheme 15) [91]. The reaction involves an addition-elimination reaction of the type discussed for the Rochow’s procedure. The organometallic reagent is, in this case, the tris(trimethylstanny1)amines which can be easily prepared from trimethyl tin chloride and lithium amide. Since the tris(trimethylstanny1)amine does not show strong basic properties, the α-deprotonation is completely suppressed thus allowing a facile preparation of tin-imines even in the case of enolizable ketones and aldehydes. An interesting feature of the tin-imines is the possibility to undergo transmetallation reactions with trialkylsilyl chlorides (e.g., chloro *tert*-butyldimethylsilane) to give the corresponding *N*-silylimine and tris(trimethyltin)onium chloride that spontaneously precipitates from the solution. Removal of this precipitate by filtration allows the preparation of almost pure solution of silylimines [91].

## 3. Importance of Schiff Bases in Organic Synthesis, Bio-Processes and Pharmaceutical Chemistry

### 3.1. Schiff Bases as Precursors of Countless Versatile Organic Processes for the Production of Intermediates/Products

**Chart 1:**
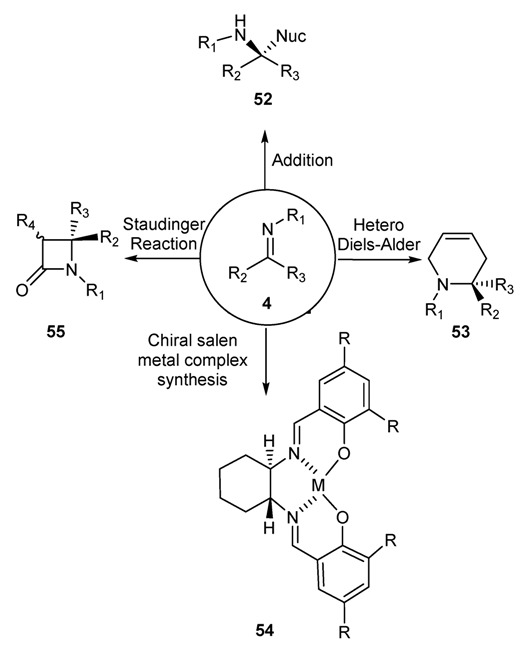
Applications of Schiff bases in organic synthesis.

As a versatile precursor for organic syntheses, we can identify, in an oversimplification, four different types of reactions in which Schiff bases have been found extremely important applications: (a) addition of organometallic reagents or hydride to C=N bond to afford compounds of structure **52**; (b) hetero Diels-Alder reaction to furnish six membered nitrogen containing heterocyclic compounds of general formula **53**; (c) skeletons for the building-up scaffolds, as the very famous *salen scaffold*, to be used as “*privileged ligand*” [92] for the formation of the corresponding *chiral salen metal complexes* **54**; (d) Staudinger reaction with ketene to furnish biologically important β-lactam ring **55** (Chart 1). It must be underlined for point (c) that we are reporting only the applications of chiral salen complexes [92,93,94,95,96]. For different complex catalysts, as salophen [97,98], or for the use of Schiff bases, different from salen backbone, we refer the interested reader to the following up-to-date survey of extremely good and dedicated reviews on the subject authored by specialists in the field [92,99,100,101,102,103,104,105].

The same criteria have been used for all the applications reported in Chart 1. Accordingly we have grouped the references reported in: (a) Reduction of C=N bond, focused on asymmetric formation of carbon-carbon bond [60,106,107,108,109]; (b) Hetero Diels-Alder reactions with the formation of heterocyclic compounds [110,111,112,113,114,115,116]; (c) Use of chiral salen metal complexes in the asymmetric synthesis [92,93,94,95,96,117,118]; (d) Staudinger reactions for the preparation of β-lactams [4,119,120,121,122]. In the following paragraphs we will emphasize the importance of imines, first discovered by the Ugo Schiff, providing the reader with relevant information highlighting the importance of Schiff bases and their applications in a wide range of organic and pharmaceutical chemistry fields.

### 3.2. Schiff Bases as Intermediate of Bio-Processes

**Scheme16:**
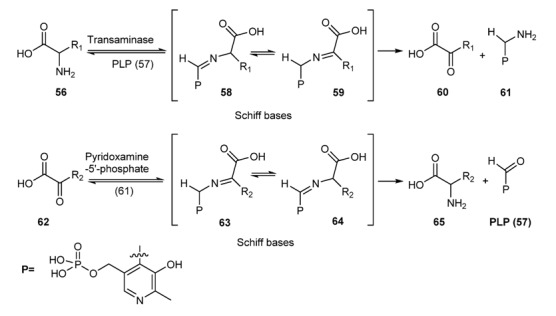
Transamination reaction through Schiff bases from amino-acid to ketoacid and *vice versa*.

The importance of Schiff bases as intermediates in bio-processes is very well established: suffice it to mention one of the very basic process of life: the transamination reaction (Scheme 16) [123].

**Scheme 17:**
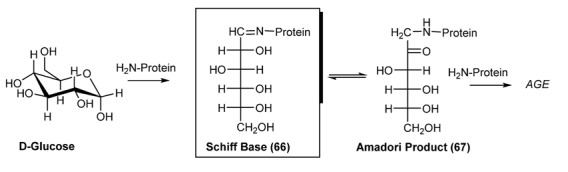
Protein glycation by glucose.

Other important bio-processes, that lately are attracting the interest of chemists and biologists, are related to the glycation of albumin that leads to the formation of important biomarkers, which are predictive of type II diabetes [124] or to the reaction between sugars and biologically relevant amines with the formation of Schiff bases. These intermediates Schiff bases **66**, in turn, evolve to **A**dvanced **G**lycation **E**ndproducts (***AGE***) through Amadori compounds (Scheme 17).

*AGEs* are involved in many pathological conditions such as cardiovascular disease [125], Alzheimer [126] and so on. Although these compounds are very important a depth discussion would take the reader into specific scientific area that goes behind the scope of this review. The following paragraphs will focus on the importance of Schiff discovery and present some examples of compounds featuring the Schiff bases as pharmaceutical garrisons.

### 3.3. Some Application of Schiff Bases in Pharmaceutical Research

**Figure 4:**
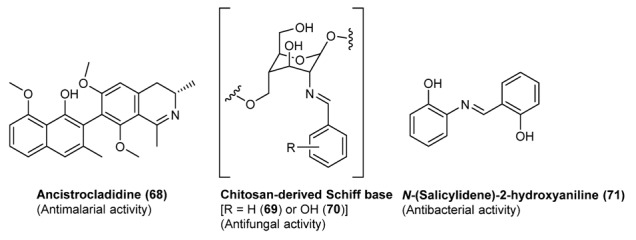
Some examples of biologically active Schiff bases.

There are numerous publications covering the use of Schiff bases in therapeutic or biological applications either as potential drug candidates or diagnostic probes and analytical tools. The activity of Schiff bases as anticancer compounds [127,128] including radioactive nuclide complexes, antibacterial [129,130,131,132,133,134,135], antifungal [25,136,137], antiviral agents [138], has been extensively studied. Moreover, Schiff bases are present in various natural, semi-synthetic, and synthetic compounds (see Figure 4 for some examples) and have been demonstrated to be essential for their biological activities [139,140].

#### 3.3.1. Antiparassitic Schiff Bases

**Scheme 18:**
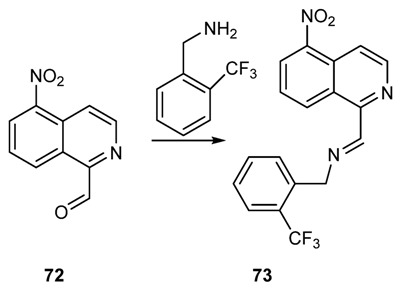
Synthesis of some 5-nitroisoquinolines Schiff bases.

Malaria is a severe morbidity of humans and other animals. It is caused by protozoa of the genus *Plasmodium*. It is initiated by a bite from an infected female *Anopheles* mosquito, which introduces the *Plasmodium* through saliva into the circulatory system. In the blood, the protists travel to the liver to mature and reproduce. Typical symptoms of malaria include fever and headache, which, in severe cases, can progress to coma and eventually death. The imino-group of Schiff bases has been shown to be valuable function to confer antimalarial activity. For example, ancistrocladidine (**68**, Figure 4), a secondary metabolite produced by plants belonging to the families *Ancistrocladaceae* and *Dioncophyllaceae*, features an imine group in its structure. The compound has shown potent activity against *P. falciparum* K1. Some novel aldimine and hydrazone isoquinoline derivatives, prepared by reacting 1-formyl-5-nitroisoquinoline with amines (Scheme 18), showed activity against a chloroquine-resistant *Plasmodium falciparum* strain (ACC Niger). In particular the corresponding Schiff base of formyl-5-nitroisoquinoline (*E*)-*N*-((5-nitroisoquinolin-1-yl)-methylene)-1-(2-(trifluoromethyl)-phenyl)methanamine (**73**, Scheme 18) showed an IC_50_ of 0.7 µg/mL against *P. falciparium* [137].

#### 3.3.2. Salicylidene Amines as Bioactive Compounds

**Figure 5:**
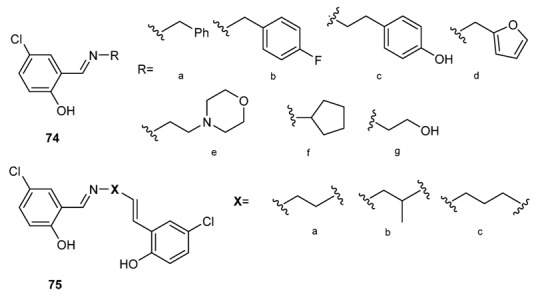
5-chloro-salicylaldehyde-Shiff bases.

**Figure 6:**
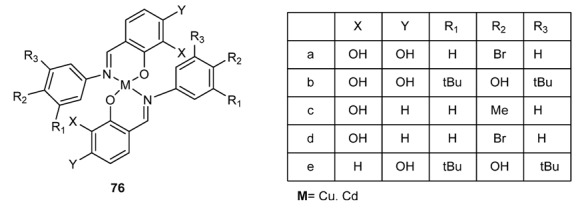
Cu(II) and Cd(II) complexes of salicylidenebenzylamines.

Salicylidenebenzylamine derivatives have been studied extensively for their biological activities [121,122,123]. Schiff base complexes derived from 4-hydroxysalicylaldehyde and amines have strong anticancer activity, e.g., against Ehrlich ascites carcinoma (EAC) [141]. *N*-(salicylidene)-2-hydroxyaniline, in turn, showed activity against *Mycobacterium tuberculosis* H37Rv [136]. The antibacterial activity of a series of 5-chlorosalicylaldehyde-Shiff bases (Scaffolds **74** and **75** Figure 5) was studied against several strains including *Escherichia coli* and *Staphylococcus aureus* [142]. Cu(II) and Cd(II) complexes **76** (Figure 6) of more highly functionalized salicylidenebenzylamines present higher activity with respect to the free molecules [143].

#### 3.3.3. Other Antibacterial Schiff Bases

**Figure 7:**
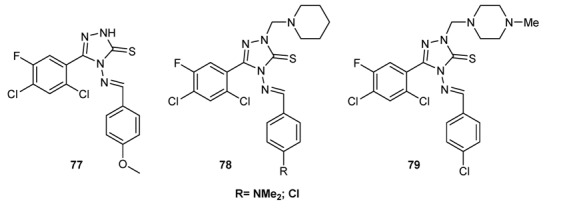
Chemical structure of 2,4-dichloro-5-ﬂuorophenyl Schiff bases.

Schiff bases characterized by a 2,4-dichloro-5-ﬂuorophenyl moiety (Figure 7) completely inhibited the growth of *S. aureus*, *E. coli*, *Pseudomonas aeruginosa*, and *Klebsiella pneumoniae* with MIC values ranging from 6.3 to 12.5 µg/mL, which are comparable to ciproﬂoxacin [134].

**Figure 8:**
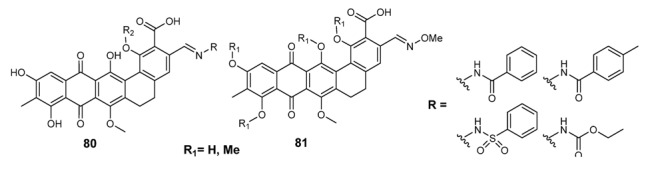
*Madurahydroxy* lactones Schiff bases.

The secondary metabolites of the plant *Actinomadura rubra*, madurahydroxylactones, have been transformed into the corresponding Schiff bases **80** (Figure 8) [144]. Madurahydroxylactone-derived compounds inhibited *in vitro B. subtilis*, *Micrococcus* *ﬂavus*, *Sarcina lutea*, and *S. aureus* giving MIC values varying from 0.2 to 3.1 µg/mL [145].

#### 3.3.4. Antifungal Schiff Bases

**Figure 9:**
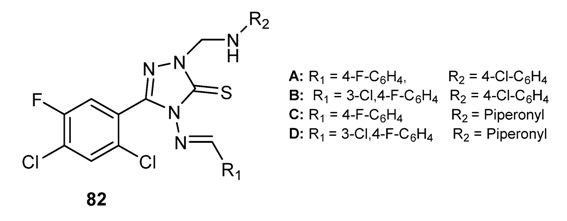
Antifungal Schiff bases derived from 2,4-dichloro-5-fluorophenyl scaffold.

**Figure 10:**
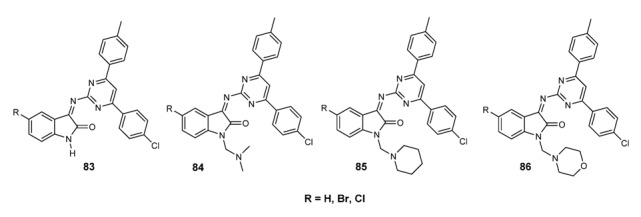
*Isatin* derived Schiff bases.

Schiff bases of chitosan **69** and **70** (See Figure 4) have shown antifungal activity against *Botrytis cinerea* and *Colletotrichum lagenarium* [140]. Imine derivatives having a 2,4-dichloro-5-ﬂuorophenyl moiety (see Figure 7) and the Schiff bases **82**, reported in Figure 9, inhibited the growth of fungal clinical isolates, such as *Aspergillus fumigatus*, *Aspergillus* *ﬂavus*, *Penicillium marneffei*, *Trichophyton mentagrophytes*. The compounds showed MIC values in the range of 6.3–12.5 µg/mL, which is comparable to that of ﬂuconazole [134]. The isatin-derived Schiff bases **83**–**86** (Figure 10) showed an interesting activity against *Microsporum audouinii* (MIC range 2.4–9.7 µg/mL) and *Microsporum gypseum* (MIC range 1.2–9.7 µg/mL) [43].

The compounds reported inhibited also *Candida albicans*, *Aspergillus niger*, *Cryptococcus neoformans*, *T. mentagrophytes*, *E. ﬂoccosum*, and *Histoplasma capsulatum* (MIC range 10–79 µg/mL [146].

#### 3.3.5. Antiviral Schiff Bases

**Figure 11:**
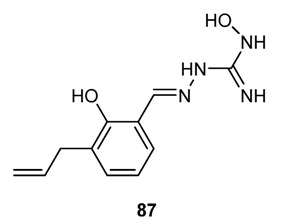
Modified 3-hydroxyguanidines antiviral Schiff base.

The Schiff bases of modified 3-hydroxyguanidines [147,148], have been prepared and tested against mouse hepatitis virus (MHV), in particular, compound **87** (Figure 11) inhibited the viral replication by 50% when used at a concentration of 3.2 µM.

**Figure 12:**
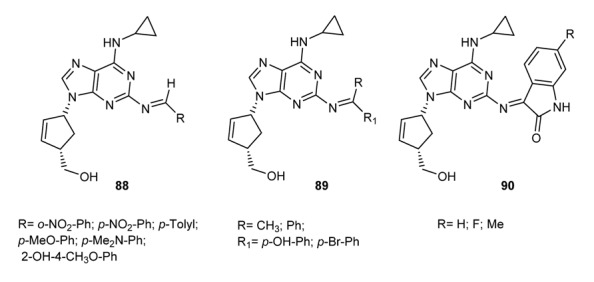
Schiff bases of abacavir.

Similarly, a set of imine derivatives of abacavir [148] have been prepared and tested for their antiviral activity. Compounds **88**–**90** in Figure 12 were highly effective against the human immunodeﬁciency virus-type 1 (HIV-1). The molecules, which are reported to be Abacavir prodrugs, showed a 50% protection of human leukemic cells (CEM) at micromolar and even nanomolar concentration (compound **87**, EC_50_ = 50 nM).

#### 3.3.6. Hybrid Structures

**Figure 13:**
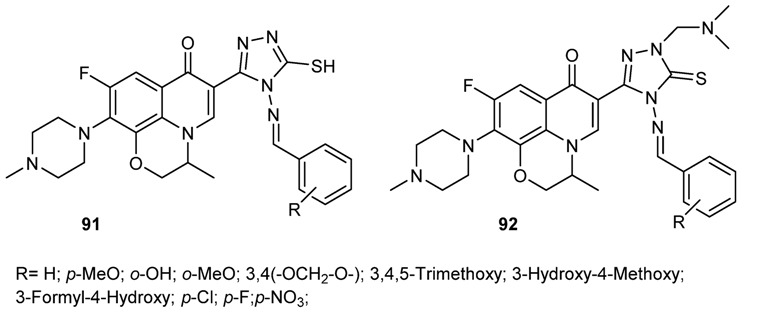
Chemical structure of hybrid fluoroquinolone-Schiff bases.

The use of hybrid structures to achieve new pharmacological activities is widely used in medicinal chemistry. In an attempt to achieve novel antitumor compounds, Schiff and Mannich bases of fluoroquinolones have been prepared and tested in cell line [149] (Figure 13). In particular compounds **92** depicted in Figure 13 showed potent activity against L1210, HL60 and CHO tumor cells in the MTT assay.

## 4. Conclusions

On typing “Schiff bases” in any chemistry database a countless number of records appears as proof of the importance of such derivatives in chemistry. They are present as reactants in umpteen synthetic organic processes, as important scaffolds in organometallic chemistry, as backbones of precious catalysts and as pharmaceutical presidiums against a series of different diseases and pathological states. According to the scope of this review we have tried to give simple headlines not pretending to account the multidisciplinary applications of Schiff Bases. The short section on *N-*metalloimines has been included because they must be considered as synthetic equivalents of the Schiff base arising from aldehydes/ketones and the simplest amine: ammonia. Our final goal has been to celebrate the name of an Italian (by adoption) founder of modern Organic Chemistry: Professor Hugo Schiff from the University of Florence.

## References

[1] Fontani M., Costa M. (2011). La Dinastia degli Schiff e l’Italia. Chimica e Industria.

[2] Chemical Heritage Foundation. http://www.chemheritage.org/discover/online-resources/chemistry-inhistory/themes/electrochemistry/berzelius.aspx.

[3] Schiff H. (1984). Mitteilungen aus dem universitatslaboratorium in Pisa: Eineneue reihe organischer basen. Justus Liebigs Ann. Chem..

[4] Tidwell T.T. (2008). Hugo (ugo) schiff, schiff bases, and a century of b-lactam synthesis. Angew. Chem. Int. Ed..

[5] Schiff H. (1866). Eine neue Reihe organischer Diamine. Justus Liebigs Ann. Chem..

[6] Shriner R.L., Hermann C.K.F., Morrill T.C., Fuson R.C. (2004). The Systematic Identification of Organic Compounds.

[7] Schiff H. (1857). Ueber die Einwirkung des Phosphorsuperchlorids auf einige anorganische Säuren. Justus Liebigs Ann. Chem..

[8] Patai S. (1970). The Chemistry of the Carbon-Nitrogen Double Bond.

[9] Tennant G., Sutherland I.O. (1979). Comprehensive organic chemistry. Comprehensive Organic Chemistry.

[10] Whitesell J.K., Winterfeldt E. (1991). Comprehensive organic synthesis. Comprehensive Organic Synthesis.

[11] Robertson G.M., Katritzky A.R., Meth-Cohn O., Rees C.W. (1995). Imines and their *N*-substituted derivatives: NH, NR and *N*-haloimines. Comprehensive Organic Functional Group Transformations.

[12] Pawlenko S., Klamann D., Hagemann H. (1980). Methoden der Organische Chemie (Houben-Weyl). Methoden der Organische Chemie (Houben-Weyl).

[13] Holm R.H., Everett J.G.W., Chakravorty A. (1966). Metal complexes of schiff bases and o-ketoamines. Prog. Inorg. Chem..

[14] Vigato P.A., Tamburini S. (2004). The challenge of cyclic and acyclic Schiff bases and related derivatives. Coord. Chem. Rev..

[15] Layer R.W. (1963). The chemistry of imines. Chem. Rev..

[16] Schiff U. (1867). Giornale di scienze naturali ed economiche. Palermo.

[17] Dobbs A.P., Rossiter S., Alan R.K., Taylor R.J.K. (2005). Imines and their *N*-substituted derivatives: NH, NR, and *N*-Haloimines. Comprehensive Organic Functional Group Transformations II.

[18] Westheimer F.H., Taguchi K. (1971). Catalysis by molecular sieves in the preparation of ketimines and enamines. J. Org. Chem..

[19] Love B.E., Ren J. (1993). Synthesis of sterically hindered imines. J. Org. Chem..

[20] Look G.C., Murphy M.M., Campbell D.A., Gallop M.A. (1995). Trimethylorthoformate: A mild and effective dehydrating reagent for solution and solid phase imine formation. Tetrahedron Lett..

[21] Billman J.H., Tai K.M. (1958). Reduction of schiff bases. II. Benzhydrylamines and structurally related compounds1a,b. J. Org. Chem..

[22] White W.A., Weingarten H. (1967). A versatile new enamine synthesis. J. Org. Chem..

[23] Liu G., Cogan D.A., Owens T.D., Tang T.P., Ellman J.A. (1999). Synthesis of enantiomerically Pure N-tert-butanesulfinyl imines (tert-butanesulfinimines) by the direct condensation of tert-butanesulfinamide with aldehydes and ketones. J. Org. Chem..

[24] Chakraborti A.K., Bhagat S., Rudrawar S. (2004). Magnesium perchlorate as an efficient catalyst for the synthesis of imines and phenylhydrazones. Tetrahedron Lett..

[25] Panneerselvam P., Nair R.R., Vijayalakshmi G., Subramanian E.H., Sridhar S.K. (2005). Synthesis of Schiff bases of 4-(4aminophenyl)-morpholine as potential antimicrobial agents. Eur. J. Med. Chem..

[26] Dalpozzo R., de Nino A., Nardi M., Russo B., Procopio A. (2006). Erbium(III) triflate: A valuable catalyst for the synthesis of aldimines, ketimines and enaminones. Synthesis.

[27] Naeimi H., Salimi F., Rabiei K. (2006). Mild and convenient one pot synthesis of Schiff bases in the presence of P2O5/Al2O3 as new catalyst under solvent-free conditions. J. Mol. Catal. A Chem..

[28] Reddelien G. (1913). Über Selbstkondensation bei Anilen. (Studien über Zinkchlorid als Kondensationsmittel III). Berichte der deutschen chemischen Gesellschaft.

[29] Varma R.S., Dahiya R., Kumar S. (1997). Clay catalyzed synthesis of imines and enamines under solvent-free conditions using microwave irradiation. Tetrahedron Lett..

[30] Schmeyers J., Toda F., Boy J., Kaupp G. (1998). Quantitative solid–solid synthesis of azomethines. J. Chem. Soc. Perkin Trans 2.

[31] Vass A., Duda S.J., Varma R.S. (1999). Solvent-free synthesis of Nsulfonylimines using microwave irradiation. Tetrahedron Lett..

[32] Tanaka K., Shiraishi R. (2000). Clean and efficient condensation reactions of aldehydes and amines in a water suspension medium. Green Chem..

[33] Andrade C.K.Z., Takada S.C.S., Alves L.M., Rodrigues J.P., Suarez P.A.Z., Brandão R.F., Rodrigo F., Soares V.C.D. (2004). Molecular sieves in ionic liquids as an efficient and recyclable medium for the synthesis of imines. Synlett.

[34] Vázquez M.Á., Landa M., Reyes L., Miranda R., Tamariz J., Delgado F. (2004). Infrared irradiation: Effective promoter in the formation of N-benzylideneanilines in the absence of solvent. Synth. Commun..

[35] Gopalakrishnan M., Sureshkumar P., Kanagarajan V., Thanusu J. (2007). New environmentally-friendly solvent-free synthesis of imines using calcium oxide under microwave irradiation. Res. Chem. Intermed..

[36] Guzen K.P., Guarezemini A.S., Órfão A.T.G., Cella R., Pereira C.M.P., Stefani H.A. (2007). Eco-friendly synthesis of imines by ultrasound irradiation. Tetrahedron Lett..

[37] Shiraishi Y., Ikeda M., Tsukamoto D., Tanaka S., Hirai T. (2011). One-pot synthesis of imines from alcohols and amines with TiO_2_ loading Pt nanoparticles under UV irradiation. Chem. Commun..

[38] Jiang L., Jin L., Tian H., Yuan X., Yu X., Xu Q. (2011). Direct and mild palladium-catalyzed aerobic oxidative synthesis of imines from alcohols and amines under ambient conditions. Chem. Commun..

[39] Huang B., Tian H., Lin S., Xie M., Yu X., Xu Q. (2013). Cu(I)/TEMPO-catalyzed aerobic oxidative synthesis of imines directly from primary and secondary amines under ambient and neat conditions. Tetrahedron Lett..

[40] Soule J.-F., Miyamura H., Kobayashi S. (2013). Selective imine formation from alcohols and amines catalyzed by polymer incarcerated gold/palladium alloy nanoparticles with molecular oxygen as an oxidant. Chem. Commun..

[41] Gnanaprakasam B., Zhang J., Milstein D. (2010). Direct synthesis of imines from alcohols and amines with liberation of H2. Angew. Chem. Int. Ed..

[42] Yuan H., Yoo W.-J., Miyamura H., Kobayashi S. (2012). Discovery of a metalloenzyme-like cooperative catalytic system of metal nanoclusters and catechol derivatives for the aerobic oxidation of amines. J. Am. Chem. Soc..

[43] Largeron M., Fleury M.-B. (2013). Bioinspired oxidation catalysts. Science.

[44] Lan Y.-S., Liao B.-S., Liu Y.-H., Peng S.-M., Liu S.-T. (2013). Preparation of imines by oxidative coupling of benzyl alcohols with amines catalysed by dicopper complexes. Eur. J. Org. Chem..

[45] Largeron M. (2013). Protocols for the catalytic oxidation of primary amines to imines. Eur. J. Org. Chem..

[46] Pickard P.L., Young C.W. (1951). Ketimines. III. ι-cyclohexylalkyl alkyl type1. J. Am. Chem. Soc..

[47] Pickard P.L., Tolbert T.L. (1961). An improved method of ketimine synthesis. J. Org. Chem..

[48] Porai-Koshits B.A., Remizov A.L. (1956). Probl. mekhanizma org. reaktsii. Chem. Abstr..

[49] Hoesch K. (1915). Eine neue Synthese aromatischer Ketone. I. Darstellung einiger Phenol-ketone. Ber. Dtsch. Chem. Ges..

[50] Hoesch K. (1917). Eine neue Synthese aromatischer Ketone. II. Künstliche Darstellung des Maclurins und ihm verwandter Ketone. Ber. Dtsch. Chem. Ges..

[51] Houben J., Fischer W. (1929). Formation of aromatic nitriles by basic hydrolysis of trichloromethyl aryl ketimines. Acidic hydrolysis yields ketones. J. Prakt. Chem..

[52] Britton E.C., Mich M., Bryner F. (1932). Method of making imides of ketones. U.S. Patent.

[53] Mosher H., Blanz J.E. (1957). Notes—Reduction of o-bromoanisole by lithium dineopentylamide. J. Org. Chem..

[54] Claisen L. (1896). Ueber eine eigenthümliche Umlagerung. Ber. Dtsch. Chem. Ges..

[55] Reddelien G. (1920). Über die Zersetzung von Anilen. (Über die katalytische Wirkungsweise von Halogenwasserstoffsäuren bei Kondensationen, II). Ber. Dtsch. Chem. Ges..

[56] Boyer J.H., Canter F.C. (1954). Alkyl and aryl azides. Chem. Rev..

[57] Langheld K. (1909). Über das Verhalten von α-Aminosäuren gegen Natriumhypochlorit. Ber. Dtsch. Chem. Ges..

[B58-molecules-18-12264] 58.In this Review other substitution to the imine-nitrogen will not be treated since they are outside of the scope of this work.

[59] Cainelli G., Panunzio M., Andreoli P., Martelli G., Spunta G., Giacomini D., Bandini E. (1990). Metallo-imines: Useful reagents in organic chemistry. Pure Appl. Chem..

[60] Barluenga J., Aznar F., Valdes C. (2004). N-trialkylsilylimines as coupling partners for Pd-catalyzed C-N bond-forming reactions: One-step synthesis of imines and azadienes from aryl and alkenyl bromides. Angew. Chem. Int. Ed..

[61] Barluenga J., Suarez-Sobrino A., Lopez L.A. (1999). Chiral heterosubstituted 1,3-butadienes: Synthesis and 4+2 cycloaddition reactions. Aldrichim. Acta.

[62] Barluenga J., Jimenez-Aquino A., Fernandez M.A., Aznar F., Valdes C. (2007). Multicomponent and one-pot synthesis of trisubstituted pyridines through a Pd-catalyzed cross-coupling/cross-coupling/cycloaddition sequence. Tetrahedron.

[63] Zhu W., Mena M., Jnoff E., Sun N., Pasau P., Ghosez L. (2009). Multicomponent reactions for the synthesis of complex piperidine scaffolds. Angew. Chem. Int. Ed..

[64] Jnoff E., Ghosez L. (1999). Asymmetric diels—alder reactions of 2-azadienes catalyzed by a chiral copper(II) complex. A general route to enantiomerically pure piperidones. J. Am. Chem. Soc..

[65] Long S., Monari M., Panunzio M., Bandini E., D’Aurizio A., Venturini A. (2011). Hetero-Diels-Alder (HDA) strategy for the preparation of 6-aryl- and heteroaryl-substituted piperidin-2-one scaffolds: Experimental and theoretical studies. Eur. J. Org. Chem..

[66] Bongini A., Panunzio M. (2006). A hetero Diels-Alder concerted vs. aldol stepwise mechanism in the cyclization of silyloxyazadienes with aldehydes: A theoretical study. Eur. J. Org. Chem..

[67] Panunzio M., Vicennati P., Pandalai S.G. (2002). From 3-Trialkylsilyloxy-2-Aza-1,3-dienes to biological interesting molecules through cyclization reactions. Recent Research Development in Organic Chemistry, Part II.

[68] Panunzio M., Bandini E., D’Aurizio A., Xia Z., Mu X. (2008). Synthesis of Venlafaxine from azadiene via a Hetero-Diels-Alder approach: New microwave-assisted transketalization and hydroxymethylation reactions. Synthesis.

[69] Bandini E., Corda G., D’Aurizio A., Panunzio M. (2010). A straightforward synthesis of conhydrine by hetero Diels-Alder strategy mediated by microwaves. Tetrahedron Lett..

[B70-molecules-18-12264] 70.We have limited our report to these metallo-imines because they have found applications in organic synthesis as Schiff-bases analogues.

[71] Lavrinovich L.I., Ignatenko A.V., Bubnov Y.N. (1992). Synthesis of functional derivatives of 20methylenecyclopentane and 2-methylenecyclohexane based in the allylborylation of imines, nitriles, isocyanates, and isothiocyanates by cycloalkenylmethyl(dipropyl)boranes. Bull. Russ. Acad. Sci. Div. Chem. Sci. (Engl. Transl.).

[72] Meller A., Maringgele W. (1968). Monomere und dimere hochhalogenierte Iminoborane. Monatsh. Chem..

[73] Evers E.C., Freitag W.O., Kriner W.A., MacDiarmid A.G. (1959). The preparation of di-n-butylboron cyanide by the interaction of di-n-butylboron chloride with trimethylsilyl cyanide1. J. Am. Chem. Soc..

[74] Hoberg H., Barluenga-Mur J. (1969). Das verhalten von dialkyl-aluminium-amiden gegenüber benzonitril. J. Organometall. Chem..

[75] Hoberg H., Barluenga J. (1970). Addition der Cα-H-bindung von *N*-aluminium-iminen und iminen an nitrile. Synthesis.

[76] Hoberg H., Barluenga J. (1970). Addition von Al[BOND]Namin-verbindungen an benzonitril. Justus Liebigs Ann. Chem..

[77] Piotrowski A., Kunicki A., Pasynkiewicz S. (1980). The reactions of tetraalkylaluminoxanes with benzonitrile. J. Organometall. Chem..

[78] Hirabayashi T., Itoh K., Sakai S., Ishii Y. (1970). Insertion reactions of diethylaluminium derivatives II. reaction of diethylaluminium dimethylamide and diethylaluminium ethanethiolate with nitriles. J. Organometall. Chem..

[79] Andreoli P., Cainelli G., Giacomini D., Martelli G., Panunzio M. (1986). A synthetic approach to azetidinones from nitriles and lithium-triethoxy aluminium hydride. Tetrahedron Lett..

[80] Andreoli P., Cainelli G., Contento M., Giacomini D., Martelli G., Panunzio M. (1998). Reaction of silylimines with ester enolates. Synthesis of *N*-unsubstituted azetidinones starting from nitriles. J. Chem. Soc. Perkin Trans. 1.

[81] Cainelli G., Giacomini D., Mezzina E., Panunzio M., Zarantonello P. (1991). *N*-metallo imines–a new approach to alpha-amino alcohols from aldehydes. Tetrahedron Lett..

[82] Cainelli G., Panunzio M., Contento M., Giacomini D., Mezzina E., Giovagnoli D. (1993). Preparation of 1,2 aminols from cyanohydrins via *N*-diisobutylaluminium imines. Tetrahedron.

[83] Chan L.-H., Roschow E.G. (1967). Syntheses and ultraviolet spectra of N-organosilyl ketimines. J. Organometall. Chem..

[84] Kruger C., Rochow G., Wannagat U. (1963). ber die einwirkung von natrium-bis-trimethylsilyl-amid auf benzophenon, benzaldehyd und benzochinon. Chem. Ber..

[85] Hart D.J., Kanai K., Thomas D.G., Yang T.K. (1983). Preparation of primary amines and 2-azetidinones via N-(trimethylsilyl)imines. J. Org. Chem..

[86] Panunzio M., Zarantonello P. (1998). Synthesis and use of N-(trimethylsilyl)imines. Org. Process Res. Dev..

[87] Cainelli G., Giacomini D., Panunzio M., Martelli G., Spunta G. (1987). β-Lactam from esters and silylimines: A revaluation. Synthesis of *N*-unsubstituted 4-Alkyl-β-lactam. Tetrahedron Lett..

[88] Gutsulyak D.V., Nikonov G.I. (2010). Chemoselective catalytic hydrosilylation of nitriles. Angew. Chem. Int. Ed..

[89] Colvin E.W., McGarry D., Nugent M.J. (1988). Silicon-assisted synthesis of β-lactams. Tetrahedron.

[90] Guillemin J.-C., Ammi L., Denis J.-M. (1988). A convenient synthesis of enolizable *N*-trialkylsilylimines using vacuum gas-solid reactions. Tetrahedron Lett..

[91] Busato S., Cainelli G., Panunzio M., Bandini E., Martelli G., Spunta G. (1991). Beta-lactams from ester enolates and metalloimines—synthesis and reactivity of tert-butyl-dimethylsilylimines. Synlett.

[92] Yoon T.P., Jacobsen E.N. (2003). Privileged chiral catalysts. Science.

[93] Cozzi P.G. (2004). Metal-Salen Schiff base complexes in catalysis: Practical aspects. Chem. Soc. Rev..

[94] Katsuki T. (2004). Unique asymmetric catalysis of *cis*-beta metal complexes of salen and its related Schiff-base ligands. Chem. Soc. Rev..

[95] Matsunaga S., Shibasaki M. (2013). Multimetallic schiff base complexes as cooperative asymmetric catalysts. Synthesis.

[96] Whiteoak C.J., Salassa G., Kleij A.W. (2012). Recent advances with pi-conjugated salen systems. Chem. Soc. Rev..

[97] Dalla Cort A., de Bernardin P., Forte G., Mihan F.Y. (2010). Metal-salophen-based receptors for anions. Chem. Soc. Rev..

[98] Szumna A. (2010). Inherently chiral concave molecules-from synthesis to applications. Chem. Soc. Rev..

[99] Das M.C., Xiang S.C., Zhang Z.J., Chen B.L. (2011). Functional mixed metal-organic frameworks with metalloligands. Angew. Chem. Int. Ed..

[100] Dhanaraj C.J., Johnson J., Joseph J., Joseyphus R.S. (2013). Quinoxaline-based Schiff base transition metal complexes: Review. J. Coord. Chem..

[101] Drozdzak R., Allaert B., Ledoux N., Dragutan I., Dragutan V., Verpoort F. (2005). Synthesis of Schiff base-ruthenium complexes and their applications in catalytic processes. Adv. Synth. Catal..

[102] Frischmann P.D., MacLachlan M.J. (2013). Metallocavitands: An emerging class of functional multimetallic host molecules. Chem. Soc. Rev..

[103] Gupta K.C., Sutar A.K. (2008). Catalytic activities of Schiff base transition metal complexes. Coord. Chem. Rev..

[104] Kumar S., Dhar D.N., Saxena P.N. (2009). Applications of metal complexes of Schiff bases-A review. J. Sci. Ind. Res. India.

[105] Lim S., Choi B., Min Y.S., Kim D., Yoon I., Lee S.S., Lee I.M. (2004). A study on the development of CVD precursors V—Syntheses and characterization of new N-alkoxy-beta-ketoiminate complexes of titanium. J. Organomet.Chem..

[106] Kobayashi S., Mori Y., Fossey J.S., Salter M.M. (2011). Catalytic enantioselective formation of C−C bonds by addition to imines and hydrazones: A ten-year update. Chem. Rev..

[107] Guizzetti S., Benaglia M. (2010). Trichlorosilane-mediated stereoselective reduction of C=N bonds. Eur. J. Org. Chem..

[108] Noble A., Anderson J.C. (2013). Nitro-mannich reaction. Chem. Rev..

[109] Verkade J.M.M., Hemert L.J.C.V., Quaedflieg P.J.L.M., Rutjes F.P.J.T. (2008). Organocatalysed asymmetric Mannich reactions. Chem. Soc. Rev..

[110] Masson G., Lalli C., Benohoud M., Dagousset G. (2013). Catalytic enantioselective [4 + 2]—cycloaddition: A strategy to access aza-hexacycles. Chem. Soc. Rev..

[111] Ali F.E., Bondinell W.E., Dandridge P.A., Frazee J.S., Garvey E., Girard G.R., Kaiser C., Ku T.W., Lafferty J.J., Moonsammy G.I. (1985). Orally active and potent inhibitors of γ-aminobutyric acid uptake. J. Med. Chem..

[112] Smith C.D., Gavrilyuk J.I., Lough A.J., Batey R.A. (2010). Lewis acid catalyzed three-component Hetero-Diels-Alder (Povarov) reaction of *N*-arylimines with strained norbornene-derived dienophiles. J. Org. Chem..

[113] Ueno S., Ohtsubo M., Kuwano R. (2009). [4 + 2] cycloaddition of o-xylylenes with imines using palladium catalyst. J. Am. Chem. Soc..

[114] Kouznetsov V.V. (2009). Recent synthetic developments in a powerful imino Diels-Alder reaction (Povarov reaction): Application to the synthesis of *N*-polyheterocycles and related alkaloids. Tetrahedron.

[115] Jørgensen K.A. (2000). Catalytic asymmetric Hetero-Diels-Alder reactions of carbonyl compounds and imines. Angew. Chem. In. Ed. Engl..

[116] Boger D.L., Weinreb S.M. (1987). Hetero Diels-Alder Methodology in Organic Synthesis.

[117] Haak R.M., Wezenberg S.J., Kleij A.W. (2010). Cooperative multimetallic catalysis using metallosalens. Chem. Commun..

[118] Jacobsen E.N. (2000). Asymmetric catalysis of epoxide ring-opening reactions. Acc. Chem. Res..

[119] Allen A.D., Tidwell T.T. (2012). New directions in ketene chemistry: The land of opportunity. Eur. J. Org. Chem..

[120] D’hooghe M., van Brabandt W., Dekeukeleire S., Dejaegher Y., de Kimpe N. (2008). Highly stereoselective synthesis of beta-lactams utilizing alpha-chloroimines as new and powerful chiral inductors. Chem. Eur. J..

[121] David O., Meester W.J.N., Bieraugel H., Schoemaker H.E., Hiemstra H., van Maarseveen J.H. (2003). Intramolecular staudinger ligation: A powerful ring-closure method to form medium-sized lactams. Angew. Chem. Int. Ed..

[122] Palomo C., Aizpurua J.M., Ganboa I., Oiarbide M. (2004). Asymmetric synthesis of beta-lactams through the Staudinger reaction and their use as building blocks of natural and nonnatural products. Curr. Med. Chem..

[123] Snell E.E., Jenkins W.T. (1959). The mechanism of the transamination reaction. J. Cell. Comp. Phys..

[124] Cohen M.P. (2013). Clinical, pathophysiological and structure/function consequences of modification of albumin by Amadori-glucose adducts. Biochimi. Biophys. Acta (BBA) Gen. Subj..

[125] Yamagishi S.-I. (2011). Role of advanced glycation end products (AGEs) and receptor for AGEs (RAGE) in vascular damage in diabetes. Exp. Geront..

[126] Grillo M.A., Colombatto S. (2008). Advanced glycation end-products (AGEs): Involvement in aging and in neurodegenerative diseases. Amino Acids.

[127] Desai S.B., Desai P.B., Desai K.R. (2001). Synthesis of some Schiff bases, thiazolidinones and azetidinones derived from 2,6-diaminobenzo1,2-d: 4,5-d’ bisthiazole and their anticancer activities. Heterocycl. Commun..

[128] Przybylski P., Huczynski A., Pyta K., Brzezinski B., Bartl F. (2009). Biological properties of Schiff bases and azo derivatives of phenols. Curr. Org. Chem..

[129] Abdel Aziz A.A., Salem A.N.M., Sayed M.A., Aboaly M.M. (2012). Synthesis, structural characterization, thermal studies, catalytic efficiency and antimicrobial activity of some M(II) complexes with ONO tridentate Schiff base N-salicylideneO-aminophenol (saphH2). J. Mol. Struct..

[130] Sinha D., Tiwari A.K., Singh S., Shukla G., Mishra P., Chandra H., Mishra A.K. (2008). Synthesis, characterization and biological activity of Schiff base analogues of indole-3-carboxaldehyde. Eur. J. Med. Chem..

[131] Vukovic N., Sukdolak S., Solujic S., Niciforovic N. (2010). Substituted imino and amino derivatives of 4-hydroxycoumarins as novel antioxidant, antibacterial and antifungal agents: Synthesis and in vitro assessments. Food Chem..

[132] Ronad P.M., Noolvi M.N., Sapkal S., Dharbhamulla S., Maddi V.S. (2010). Synthesis and antimicrobial activity of 7-(2-substituted phenylthiazolidinyl)-benzopyran-2-one derivatives. Eur. J. Med. Chem..

[133] Amin R., Krammer B., Abdel-Kader N., Verwanger T., El-Ansary A. (2010). Antibacterial effect of some benzopyrone derivatives. Eur. J. Med. Chem..

[134] Karthikeyan M.S., Prasad D.J., Poojary B., Bhat K.S., Holla B.S., Kumari N.S. (2006). Synthesis and biological activity of Schiff and Mannich bases bearing 2,4-dichloro-5-fluorophenyl moiety. Bioorg. Med. Chem..

[135] Saravanan G., Pannerselvam P., Prakash C.R. (2010). Synthesis and anti-microbial screening of novel Schiff bases of 3-amino-2-methyl quinazolin 4-(3H)-one. J. Adv. Pharm. Technol. Res..

[136] De Souza A.O., Galetti F.C.S., Silva C.L., Bicalho B., Parma M.M, Fonseca S.F.,  Marsaioli A.J., Trindade A.C.L.B., Freitas-Gil R.P., Bezerra F.S. (2007). Antimycobacterial and cytotoxicity activity of synthetic and natural compounds. Quim. Nova..

[137] Rathelot P., Vanelle P., Gasquet M., Delmas F., Crozet M.P., Timon-David P., Maldonado J. (1995). Synthesis of novel functionalized 5-nitroisoquinolines and evaluation of in vitro antimalarial activity. Eur. J. Med. Chem..

[138] Jarrahpour A., Khalili D., de Clercq E., Salmi C., Brunel J.M. (2007). Synthesis, antibacterial, antifungal and antiviral activity evaluation of some new bis-Schiff bases of isatin and their derivatives. Molecules.

[139] Bringmann G., Dreyer M., Faber J.H., Dalsgaard P.W., Staerk D., Jaroszewski J.W. (2004). Ancistrotanzanine C and related 5,1’ and 7,3’ -coupled naphthylisoquinoline alkaloids from Ancistrocladus tanzaniensis. J. Nat. Prod..

[140] Guo Z., Xing R., Liu S., Zhong Z., Ji X., Wang L. (2007). Antifungal properties of Schiff bases of chitosan, *N*-substituted chitosan and quaternized chitosan. Carbohydr. Res..

[141] Wu Z.S., Lu Z.P., Yen Z.H. (1993). Synthesis, characterization and antifungal activity of glycylglycine Schiff base complexes of 3d transition metal ions. Trans. Met. Chem..

[142] Shi L., Ge H.M., Tan S.H., Li H.Q., Song Y.C., Zhu H.L. (2007). Synthesis and antimicrobial activities of Schiff bases derived from 5-chloro-salicylaldehyde. Eur. J. Med. Chem..

[143] Golcu A., Tumer M., Demirelli H., Wheatley R.A. (2005). Cd(II) and Cu(II) complexes of polydentate Schiff base ligands: Synthesis, characterization, properties and biological activity. Inorg. Chim. Acta.

[144] Paulus E.F., Dornberger K., Werner W., Fenske D. (1994). Madurahydroxylactone. Acta Crystallogr.

[145] Heinisch L., Roemer E., Jutten P., Haas W., Werner W., Mollmann U. (1999). Semisynthetic derivatives of madurahydroxylactone and their antibacterial activities. J. Antibiot..

[146] Pandeya S., Sriram D., Nath G., de Clercq E. (1999). Synthesis and antimicrobial activity of Schiff and Mannich bases of isatin and its derivatives with pyrimidine. IL Farmaco.

[147] Wang P.H., Keck J.G., Lien E.J., Lai M.M.C. (1990). Design, synthesis, testing and quantitative structure–activity relationship analysis of substituted salicylaldehyde Schiff bases of 1-amino-3hydroxyguanidine tosylate as new antiviral agents against coronavirus. J. Med. Chem..

[148] Sriram D., Yogeeswari P., Myneedu N.S, Saraswat V. (2006). Abacavir prodrugs: Microwave-assisted synthesis and their evaluation of anti-HIV activities. Bioorg. Med. Chem. Lett..

[149] Hu G., Wang G., Duan N., Wen X., Cao T., Xie S., Huang W. (2012). Design, synthesis and antitumor activities of fluoroquinolone C-3 heterocycles (IV): S-triazole Schiff–Mannich bases derived from ofloxacin. Acta Pharm. Sinica B.

